# Cellular therapy to target neuroinflammation in amyotrophic lateral sclerosis

**DOI:** 10.1007/s00018-013-1480-4

**Published:** 2013-10-08

**Authors:** Federica Rizzo, Giulietta Riboldi, Sabrina Salani, Monica Nizzardo, Chiara Simone, Stefania Corti, Eva Hedlund

**Affiliations:** 1 Dino Ferrari Centre, Neuroscience Section, Department of Pathophysiology and Transplantation, University of Milan, Neurology Unit, IRCCS Foundation Ca’Granda Ospedale Maggiore Policlinico, 20135 Milan, Italy; 2 Department of Neuroscience, Karolinska Institutet, Retzius v. 8, 17177 Stockholm, Sweden

**Keywords:** Amyotrophic lateral sclerosis, Astrocytes, Microglia, T-lymphocytes, Motor neuron

## Abstract

Neurodegenerative disorders are characterized by the selective vulnerability and progressive loss of discrete neuronal populations. Non-neuronal cells appear to significantly contribute to neuronal loss in diseases such as amyotrophic lateral sclerosis (ALS), Parkinson, and Alzheimer’s disease. In ALS, there is deterioration of motor neurons in the cortex, brainstem, and spinal cord, which control voluntary muscle groups. This results in muscle wasting, paralysis, and death. Neuroinflammation, characterized by the appearance of reactive astrocytes and microglia as well as macrophage and T-lymphocyte infiltration, appears to be highly involved in the disease pathogenesis, highlighting the involvement of non-neuronal cells in neurodegeneration. There appears to be cross-talk between motor neurons, astrocytes, and immune cells, including microglia and T-lymphocytes, which are subsequently activated. Currently, effective therapies for ALS are lacking; however, the non-cell autonomous nature of ALS may indicate potential therapeutic targets. Here, we review the mechanisms of action of astrocytes, microglia, and T-lymphocytes in the nervous system in health and during the pathogenesis of ALS. We also evaluate the therapeutic potential of these cellular populations, after transplantation into ALS patients and animal models of the disease, in modulating the environment surrounding motor neurons from pro-inflammatory to neuroprotective. We also thoroughly discuss the recent advances made in the field and caveats that need to be overcome for clinical translation of cell therapies aimed at modulating non-cell autonomous events to preserve remaining motor neurons in patients.

## Introduction

Amyotrophic lateral sclerosis (ALS) is a fatal neurodegenerative disorder characterized by the selective and progressive deterioration of cortical, brainstem, and spinal cord motor neurons. This pathological process clinically results in progressive muscle weakness and atrophy, spasticity, respiratory failure, and finally death [1]. ALS is dominantly inherited in 5–10 % of cases [termed “familial ALS” (fALS)], but approximately 90 % of ALS patients have no apparent family history and are often termed “sporadic” (sALS) [2, 3]. However, the distinction between familial and sporadic ALS is somewhat artificial since there is a strong genetic component in sALS [4–6]. Over the last decades, numerous ALS-causing or ALS-associated genes have been identified [4, 7–14]. In particular, 20 % of fALS patients are characterized by toxic gain-of-function mutations in superoxide dismutase 1 (SOD1) [4, 11, 13]. A massive intronic hexanucleotide repeat in the C9ORF72 gene is, to date, the most common cause of fALS (20–40 %, depending on the population) and sALS (10 %) [7, 12]. It is unclear why the GGGGCC_*n*_ expansion repeats in this gene, with as far unknown function, lead to ALS. ALS is also caused by mutations in the genes encoding the DNA/RNA-binding proteins: TAR DNA-binding protein 43 (TDP43) and fused in sarcoma/translocated in liposarcoma (FUS), the ubiquitin-like protein Ubiquilin 2 [8], angiogenin, the actin-binding protein profilin [13], valosin-containing protein (VCP) (or transitional endoplasmic reticulum ATPase), and optineurin [4, 9, 10]. Riluzole is currently the only drug clinically proven to improve survival of ALS patients, but its action is not clearly understood and survival is only extended 2–3 months, with little functional improvement [15].

**Fig. 1:**
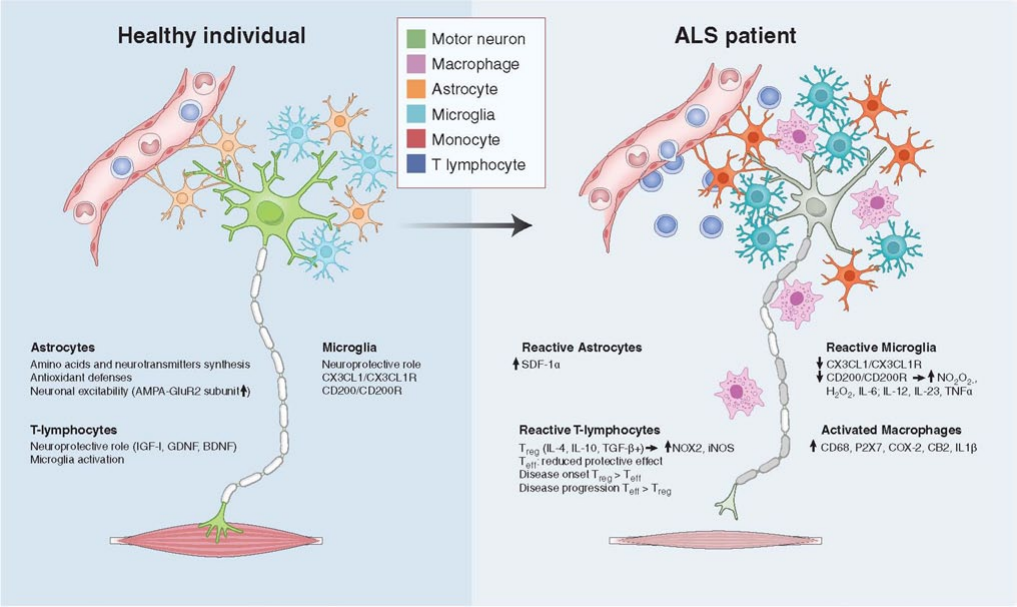
Cross-talk between motor neurons, astrocytes, and immune cells (including microglia, T lymphocytes, and macrophages) in a healthy individual (**a**) and an ALS patient (**b**)

While motor neurons are the main target in ALS, non-neuronal cells significantly contribute to motor neuron dysfunction and death. Indeed, mice chimeric for mutant SOD1 (mSOD1) show prolonged life span. Here, wild-type motor neurons in close proximity to mSOD1-containing non-neuronal cells degenerated in an ALS-like way [16]; while mSOD1-containing motor neurons surrounded by wild-type non-neuronal cells appeared less affected. Interestingly, removal of mSOD1 from motor neurons delayed the onset and early progression of disease, but did not affect the late progression phase [17, 18]. ALS is not classified as an auto-immune disorder, but neuroinflammatory processes elicited by microglia and astrocytes appear to play fundamental roles in ALS pathology [19–41]. Indeed, removal of mSOD1 from either microglial cells or astrocytes in fALS mice prolonged the late progression of the disease [17, 40]. It is apparent from these studies that in ALS there is communication between motor neurons, astrocytes, and distinct immune cells with consequent activation at sites of neuronal injury. Neurotoxic signaling from motor neurons appears to stimulate cells to produce reactive oxygen species and pro-inflammatory cytokines, causing motor neuron stress, cell damage, and initiating a self-propagating cycle of progressive cell death [18, 41, 42]. Hence, activated cells shift from an anti-inflammatory and neuroprotective role to one that is pro-inflammatory and neurotoxic. The cell populations that participate in this neuroinflammatory reaction include microglia, astrocytes, and T lymphocytes ([18, 28, 41, 42]; Fig. 1). Microglia, key players in brain damage and disorders, can play a deleterious or beneficial role based on their intrinsic characteristics, their interactions with the microenvironment, and the presence of pathogens [18]. The behavior of microglia is closely related to the action of T lymphocytes and astrocytes. Astrocytes are of neuroectodermal origin and do not belong to the immune system, but may take part in the immune response, particularly in pathological conditions involving neuronal damage [43]. In ALS, astrocytes acquire toxic attributes and subsequently contribute to motor neuron degeneration [32, 36, 38, 39]. Infiltrating T lymphocyte subpopulations appear to contribute to an endogenous neuroprotective response in ALS by increasing the protective capacity of microglia and limiting their toxic responses [26–29].

Neuroinflammation plays an important role in disease progression also in Alzheimer’s and Parkinson disease [44, 45]. Thus, finding appropriate tools to effectively target neuroinflammation could be of vital importance to delay disease progression in several neurodegenerative disorders. Multiple compounds with anti-inflammatory properties were previously tested in fALS mSOD1 animal models with positive outcomes in terms of weight loss, survival, and functional performances. The compounds tested include: minocycline, a broad-spectrum tetracycline antibiotic, which decreases the ability of T cells to contact microglia, subsequently impairing cytokine production [46–48]; thalidomide and lenalidomide, which modulate the production of inflammatory cytokines TNF-alpha, Il-1, IL-6, IL10, and IL-12 [49–51]; celecoxib, a sulfonamide nonsteroidal anti-inflammatory drug (NSAID) and selective inhibitor of cyclooxygenase 2 (COX-2), which results in reduced production of inflammatory prostaglandins [52]; rofecoxib plus creatinine, where rofecoxib is a NSAID and COX-2 inhibitor, which causes an increased risk of heart attack and stroke with long-term usage [53]; sulindac, a NSAID, which inhibits COX-2 [54] and the anti-diabetic drug pioglitazone, which is an agonist of the peroxisome proliferator-activated receptor gamma with anti-inflammatory effects [55, 56]. Based on the positive results in ALS mouse models, clinical trials were conducted with minocycline [57], thalidomide [58], celecoxib [59], cyclophosphamide [60, 61], and pioglitazone [62, 63]. Unfortunately, none of the trials resulted in an improved outcome for ALS patients. The minocycline trial in fact even showed negative results for treated patients [64]. There could be several reasons for the failure of these clinical trials: (1) Moderately positive results from animal studies with presymptomatic ALS mice do not necessarily translate into successful clinical trials when the same compound is given to patients with advanced stages of ALS. This is not a reflection of any lack of utility of mSOD1 fALS mouse models, but rather illustrates that a small positive effect in these mice should predict low success in humans. Nonetheless, it will be of high relevance to validate therapies in additional genetic models of ALS, when such are available. (2) The patient selection for the trials might not have been optimal. While neuroinflammation occurs in ALS patients independent of the cause of the disease and thus should be a shared target, it is however likely that patients will respond differently to certain therapies depending on their progression rate and disease state when receiving the treatment. Thus, understanding when and which patients would benefit more or less from specific therapies is a crucial, but challenging task. (3) The use of suboptimal dosing regimens in clinical trials could give false-negative results.

Neuroinflammation may be better modulated through cell-based therapies. Here, we discuss the potential of cellular therapy to modulate the activity of non-neuronal cells, including astrocytes, microglia, and T-lymphocytes, which contribute to the disease progression of ALS. Dysfunctional non-neuronal cells could be replaced by cell transplantation to modulate the inflammatory environment surrounding motor neurons. These pathways are not currently controlled by pharmaceuticals used in clinics.

## Astrocytes

**Fig. 2:**
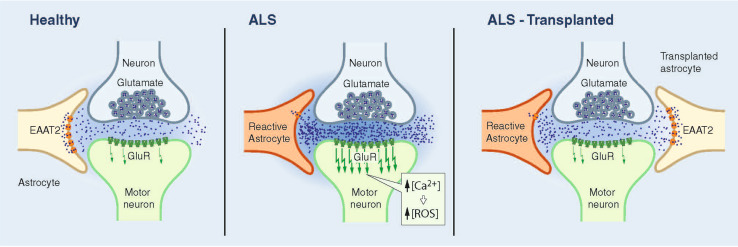
Tripartite synapse in health and in ALS: CNS functions depend on a network that includes both pre- and post-synaptic terminals of neurons and astrocytes. *Left* In a healthy individual, astrocytes take up glutamate, which is released into the synaptic cleft, through sodium-dependent excitatory amino acid transporter-1 (EAAT1) and -2 (EAAT2; GLT1 in mice). *Middle* In ALS patients and rodent fALS models, EAAT2 expression is reduced in astrocytes in the motor cortex and spinal cord, which could cause an accumulation of excitotoxic levels of extracellular glutamate and subsequently increase the neuronal intracellular calcium concentration and initiate cascades that regulate motor neuron death. *Right* Transplantation of healthy astrocytes expressing EEATs into an ALS host could sequester excess glutamate from the synapse and decrease excitotoxicity

Astrocytes are the most abundant cellular population in the CNS and outnumber their neuronal counterparts approximately tenfold. In physiological conditions, astrocytes play key housekeeping roles offering structural, metabolic, and trophic support to motor neurons [65]. They are fundamental to the catabolism and synthesis of amino acids and neurotransmitters in the CNS. Astrocytes represent a glycogen reserve and are very important in the antioxidant protection of the brain, controlling the susceptibility of neurons to noxious stimuli [65–67]. During development, astrocytes establish tight associations with endothelial cells that form blood vessels and with neurons as these establish new synapses and organize circuits. The interaction between these cell types aids in blood brain barrier (BBB) development and maintenance and particularly in the control of cerebral blood flow [68, 69]. Astrocytes also influence neuronal excitability, regulate neurotransmitter concentrations, and integrate and process synaptic information. Astrocytes are involved in the exchange of information between neurons. Thus, in addition to the traditionally “bipartite” communication between the pre- and post-synaptic terminals of neurons, CNS functions depend on a network defined as the *“*tripartite synapse” that includes both neurons and astrocytes ([70]; Fig. 2). Indeed, astrocytes actively control the structural and functional plasticity of synapses throughout the CNS [70–74].

In pathological conditions, such as ALS, reactive astrogliosis occurs, in which astrocytes are modified both molecularly and morphologically [21–25, 75]. The astrocytes modify their phenotype, adopting a cellular morphology characterized by hypertrophic nuclei and cell bodies with distinct long and thick processes with increased glial fibrillary acidic protein (GFAP). Their morphological activation is accompanied by changes in expression levels and/or type of markers such as cytoskeletal proteins, cell surface and matrix molecules, growth factors, and cytokines [76]. In addition to GFAP, precursor markers including vimentin, nestin, radial glial cell marker-2, and brain lipid binding protein are up-regulated, mostly in the initial phase after damage [75, 77]. The morphological modifications of astrocytes are associated with physiological changes, including in the molecules that astrocytes secrete; thus, cells shift from a “neuroprotective” to a “neurodegenerative” role towards motor neurons, and thus are potential therapeutic targets [78]. A population of astrocytes derived from SOD1^G93A^ fALS rats present an aberrant phenotype (referred to as “AbA cells”) with increased proliferative capacity. These AbA cells display astrocytic markers, particularly S100β and connexin 43, but lack the excitatory amino acid transporter (GLT-1) and the glial progenitor marker NG2 glycoprotein. Moreover, these AbA cells secrete soluble factors that induce motor neuron death [78, 79]. There appears to be a complex interaction between astrocytes and motor neurons in ALS, leading to astrocyte activation. Degenerating motor neurons release fibroblast growth factor 1 that activates astrocytes, which in turn increases their oxidation levels and the production of pro-inflammatory/apoptotic factors including nerve growth factor (NGF) [38, 67, 78, 79]. Astrocytes overexpressing mSOD1 release soluble factors that trigger degeneration of motor neurons, with motor neurons overexpressing mSOD1 being particularly vulnerable. The toxic effect of mSOD1 astrocytes appears specific to motor neurons, as interneurons, GABAergic, and dorsal root ganglia neurons are not affected [31, 32, 36, 38, 39]. Engraftment of mSOD1-astrocyte progenitors (which mature into astrocytes) into wild-type mice induced motor neuron death in the host ([80]; Table 1). Mechanistically, astrocytes seem to activate NOX2 to synthesize superoxide. Apocynin, a NOX2 inhibitor, prevented motor neuron loss caused by SOD1-mutated astrocytes in vitro [38]. The precursor form of NGF, produced by reactive astrocytes in response to peroxynitrite, also induces degeneration of motor neurons through the p75 neurotrophin receptor and its co-receptor on motor neurons [67, 78, 79]. While astrocyte transplantation appears less technically challenging than replacing motor neurons with their long axons and synaptic connections at multiple foci, these data pose important questions regarding the feasibility of therapeutic astrocyte engraftment. If there is already marked astrocyte proliferation, is it still useful to introduce additional astrocytes, even with a healthy phenotype? It is still not known whether donor astrocytes could be negatively influenced by the toxic host environment and by activation signals from degenerating motor neurons, and if healthy transplanted cells can turn into aberrant astrocytes. Encouragingly, initial studies indicate that wild-type rodent astrocytic precursors show some resistance to developing pathological features and do not show signs of ubiquitination in an ALS host environment [67]. On the other hand, mSOD1 astrocytes induced ubiquitination of host wild-type motor neurons [80], providing insight as to why mSOD1 non-neuronal cells can induce damage to wild-type motor neurons, as described in chimeric fALS mouse experiments [16]. Mutant SOD1 misfolding seems to be essential, and mSOD1 aggregates appear to propagate in a prion-like manner from neuronal cell to neuronal cell without cellular contact, but with the extracellular release of aggregates [81]. Misfolded SOD1 can induce misfolding of natively structured wild-type SOD1 through a direct protein–protein interaction [82]. Thus, astrocytes could contribute to a prion-like spread of misfolded proteins leading to motor neuron loss. Interestingly, astrocytes generated from neural progenitor cells that were isolated from post-mortem tissue from both fALS and sALS patients were similarly toxic specifically to wild-type motor neurons [34]. Consequently, it appears that both fALS and sALS astrocytes secrete factors that are toxic to motor neurons, or alternatively do not provide factors needed for motor neuron survival, resulting in cell death. Furthermore, shRNA suppression of SOD1 in sALS astrocytes could confer significant motor neuron protection from ALS astrocyte-derived toxicity, demonstrating that not only mSOD1, but also wild-type SOD1 could be involved in the pathogenesis of sALS [34]. The finding that neural progenitor cell-derived astrocytes from sALS postmortem patients can be toxic questions the feasibility of autologous cell transplantation with reprogrammed cells [induced pluripotent stem cells (iPSCs)] from the patient. However, to clearly understand the mechanisms and level of toxicity of sALS astrocytes, it remains to be investigated if sALS and fALS astrocytes are equally detrimental to motor neurons upon activation [in response to an ALS environment or to lipopolysaccharide (LPS)] and their secretion pattern in such a context.

**Table 1:** Recent advancements in astrocyte [glial-restricted precursors (GRP)] transplantation for ALS

Transplanted cells	Host	Outcome	References
Wild-type rat or mouse GRPs	SOD1^G93A^ rats	Reduced microgliosis	[67]
		Extended survival and disease duration. Improved motor functions	
GLT1^−/−^ rat or mouse GRPs	SOD1^G93A^ rats	No extension of disease duration	
Human fetal neural tissue GRPs	SOD1^G93A^ mice	No demonstrated motor neuron protection	[29]
		No therapeutic benefits on disease	
Wild-type (B6SJL) GRPs	Wt rats	Focal motor neuron protection	[64]
mSOD1 mouse GRPs	Wt rats	Focal motor neuron degeneration	
		Declined forelimb motor and respiratory physiological functions	

Astrocytes have a close physical relationship with motor neurons through elaborate end-feet processes that are in contact with synapses and microvessel walls (Fig. 1). The tripartite synaptic network between motor neurons and glia will likely need to be reproduced to achieve an optimal therapeutic benefit of grafted cells in ALS. It is not clear how to achieve this goal, which presumably would involve “convincing” dysfunctional disease-astrocytes to physically “give up” their established connections with motor neurons and be replaced by healthy astrocytes, if not occurring spontaneously (Fig. 2). Transplantation of lineage-restricted astrocyte precursors, a.k.a. glial-restricted precursors (GRPs), to enrich for normal astrocytes in SOD1^G93A^ mice, appeared to promote motor neuron protection locally ([37, 80, 83]; Table 1) and improve survival [83]. Cells were transplanted around cervical spinal cord respiratory motor neuron pools, the loss of which results in respiratory failure and death in ALS, and transplanted GRPs showed robust survival up to 3 months post-transplantation. The vast majority of transplanted cells were localized near the sites of injection in both white and gray matter regions of the cervical spinal cord [37, 83]. Transplanted cells also migrated to adjacent white matter regions both rostrally and caudally, although the number of cells gradually declined as the distance from the injection site increased. It is likely that the focal effects of donor astrocytes were limited due to their relatively restricted capacity to migrate. Consequently, to treat ALS using astrocytes, it would be necessary to use multiple injection sites to obtain therapeutic benefit or to focus the grafting sites to specific CNS nuclei, such as the phrenic nerve, to preserve certain crucial functions. Strategies aimed at enhancing transplanted astrocyte or astrocyte precursor migration in the CNS should improve their therapeutic potential. Therefore, it could be beneficial to genetically engineer donor cells to overexpress the polysialylated neural cell adhesion molecule (PSA-NCAM), which seems to regulate precursor cell migration during CNS development [84]. When this approach was used on adult macaque Schwann cells, their migration was stimulated and cellular interactions with reactive astrocytes were promoted at injury sites without negatively interfering with their myelination properties [85]. Furthermore, several components of the chemokine CXC motif receptor (CXCR) system could be modulated in donor astrocytes to improve migration. For example, the CXCL12/CXCR4 signaling system regulates the correct migration of neuronal cell populations in the rodent brain [86, 87]. Neural cell migration is also activated by stromal cell-derived factor 1α/chemokine receptor 4 (SDF-1α/CXCR4) [88, 89] and neuronal cells express CXCR4, the cognate receptor for SDF-1α. Human neuronal cells migrate toward sites of damage, where neighboring astrocytes up-regulate the inflammatory SDF-1α [88]. Modulation of these factors in astrocytes could increase migration towards sites of neuronal damage in response to specific cytokine gradients.

To identify the astrocyte or astrocyte precursor population most suitable as a cellular source for transplantation in ALS, these cells should be studied as heterogeneous populations, rather than a homogeneous group. Astrocytes differ in morphology, metabolism, developmental origin, gene expression profile, physiological aspects, and functions [90]. The major historical distinctions of astrocyte subtypes were based on morphological and antigenic criteria. Positionally, distinguished astrocytes residing in the grey matter were defined as protoplasmic and those situated in the white matter were called fibrous [91–94]. Recent data have further highlighted the positional heterogeneity of astrocyte precursors in the spinal cord. Three positionally distinct subtypes of astrocytes in the white matter could be distinguished based on their combinatorial expression of Reelin and Slit1. Loss- and gain-of-function experiments indicate that Pax6 and Nkx6.1 control the identities of these astrocytes [95]. Furthermore, astrocytes appear to be allocated to spatial domains in the mouse brain and spinal cord in accordance with their embryonic site of origin in the ventricular zone. The domain-specific depletion of astrocytes in the ventral spinal cord resulted in abnormal synaptogenesis, which could not be rescued by astrocytes migrating from other regions [96]. Consequently, the “positional identity” of astrocytes appear to reflect a unique “functional identity”. Importantly, astrocytes obtained from various brain regions differ in their capacities to stimulate neuronal migration, survival, growth, and uptake of extracellular glutamate, aspects that are critical in the pathology of ALS [90, 97, 98]. These observations suggest that specific subpopulations of astrocytes could show promise for cellular transplantation in ALS, depending on the site of motor neuron loss. Pre-selection of these subpopulations is very likely to influence the ability to recreate complex interactions between motor neurons and astrocytes, which are altered in ALS. Moreover, differences in surface glycoprotein expression correspond to variations in astrocyte-neuron or astrocyte–astrocyte interactions [90]. Genetic modification of these expression profiles could be an important strategy for restoring astrocyte-motor neuron interactions in ALS.

It is not yet fully understood what factors affect the integration of grafted astrocyte precursors and mature astrocytes. The host environment could influence graft integration. If the host retains some instructive capacity, grafted cells may attain slightly different functions depending on the injection site. There appears to be species differences in cell maturation after transplantation, with mouse precursors differentiating into mature astrocytes more robustly than human precursors ([37, 83]; Table 1). This propensity could be due to instructive differences between species, where a rodent host cannot fully instruct human cells to differentiate. Astrocyte precursors do not appear to have the same benefit as mature astrocytes on nearby motor neurons in ALS rodents, indicating that a replacement of connectivity with motor neurons is needed rather than just trophic support ([37, 83]; Table 1). Therefore, strictly controlled differentiation is necessary before in vivo cell transplantation, especially when astrocytes derived from iPSCs, including embryonic stem cells (ESCs) and induced iPSCs, are used as cellular sources.

**Fig. 3:**
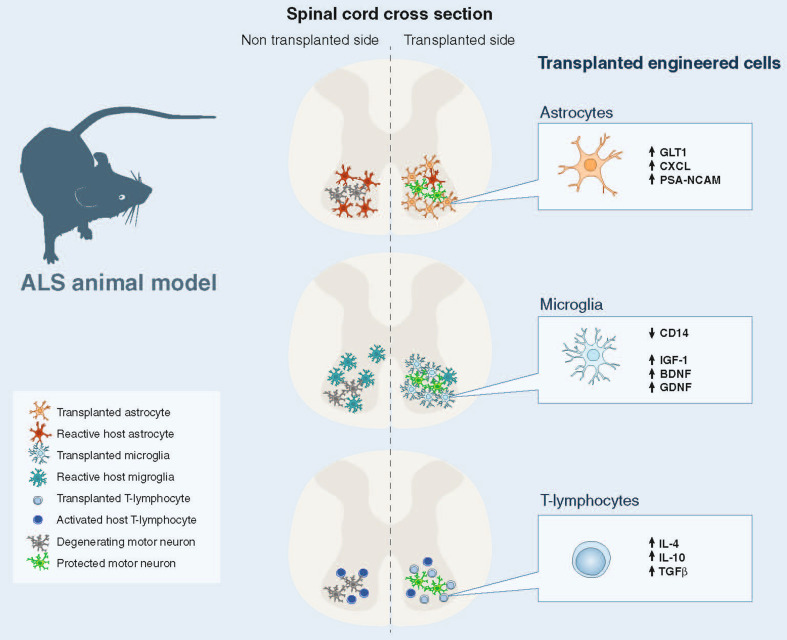
Transplantations of wild-type or genetically engineered astrocytes, microglia, and T-lymphocytes are feasible and potential future therapeutic approaches for ALS

In the absence of damage, astrocytes uptake glutamate, which is released into the synaptic cleft, through sodium-dependent excitatory amino acid transporter-1 (EAAT1) and -2 (EAAT2; GLT1 in mice). Normally, astrocytes protect motor neurons from excitotoxicity, stimulating AMPA-GluR2 subunit upregulation and the generation of receptors that are impermeable to calcium, as demonstrated in co-cultures of motor neurons and astrocytes. Overexpression of human mSOD1 in astrocytes abolished their capacity to induce GluR2 up-regulation [99]. Transplanted mSOD1-GRPs, which induced wild-type motor neuron degeneration, reduced GLT-1 transporter expression in wild-type animals in a non-cell autonomous manner [80]. In ALS patients and rodent models, EAAT2 expression is reduced in astrocytes in the motor cortex and spinal cord, which could cause an accumulation of excitotoxic levels of extracellular glutamate and subsequently increase the neuronal intracellular calcium concentration and initiate cascades that regulate motor neuron death ([99–103]; Fig. 2). The efficacy of rodent astrocyte precursors engraftment on motor neuron preservation, while modest, appeared partly mediated by the replacement of astrocyte GLT-1 (Table 1) [83]. Disappointingly, human GRPs did not slow the loss of intra-spinal GLT-1 protein levels when transplanted in mutant SOD1 mice and showed no effect on motor neuron survival [37]. Overall, these data suggest that GLT-1 expression plays an important role in the potential therapeutic effect of astrocytes. It will be interesting to see if astrocytes engineered to overexpress GLT-1 show greater therapeutic effects (Fig. 3). It is noteworthy that SOD1^G93A^ astrocyte-induced motor neuron death seems in part mediated by host microglial activation [80]. In addition, wild-type rodent GRP transplantation led to the reduction of microgliosis, implicating a positive anti-inflammatory effect of wild-type astrocytes [37].

Given the numerous examples of astrocyte dysfunction in ALS, targeting astrocytes and astrocyte replacement are promising therapeutic approaches that have shown positive results in fALS animal models (Table 1). The feasibility to replace or integrate endogenous astrocytes by transplanting GRPs derived from the CNS has been examined; however, further pre-clinical studies are needed to avoid unexpected pitfalls at the clinical level. In general, human primary CNS astrocyte precursors show robust survival, efficient differentiation, and lack of tumor formation, features that are promising for their translational potential in future ALS treatments. It is now important to determine the therapeutic profile of astrocytes derived from ESCs and iPSCs.

Another glial cell type, NG2^+^ cells (marked by the nerve-glia factor 2 proteoglycan antibody) might be of great importance in the pathogenesis and treatment of ALS. NG2^+^ cells respond rapidly to any disturbances in the CNS environment by modifying their morphology and dividing. In doing so, they contribute to a changing cellular environment by producing new oligodendrocytes, astrocytes, and neurons [104]. In ALS, NG2^+^ cells exhibit enhanced proliferation in regions of motor neuron degeneration [105, 106]. It was initially reported that a small percentage of NG2^+^ cells differentiated into astrocytes in response to proinflammatory cytokine signaling [92]. However, a recent fate-mapping analysis of NG2^+^ cells using PDGFαR-CrER transgenic mice demonstrated that NG2^+^ cells remained committed to an oligodendrocyte lineage in adult wild-type mice as well as in symptomatic SOD1^G93A^ fALS mice [106]. However, these recently formed oligodendrocytes in the fALS mice failed to fully mature and did not remyelinate axons. Such myelination defects were found also in postmortem spinal cord and motor cortex from ALS patients [107]. Importantly, inactivation of mSOD1 specifically in NG2^+^ cells delayed disease onset in mice and increased their survival, suggesting that oligodendrocytes are a potential therapeutic target in ALS [107]. NG2^+^ cells are also modulated in other neurodegenerative diseases, including Alzheimer’s and Parkinson disease [108], and following stroke and acute CNS trauma. NG2^+^ cells appear to show large lineage plasticity in response to different disorders and injuries [109, 110]. It is therefore possible that NG2^+^ cells could be modulated in vivo to generate cell types other than oligodendrocytes that could also be beneficial in ALS.

## Microglia

Microglia are resident immune cells in brain and spinal cord, originating from hematopoietic stem cells (HSC), particularly from precursors of the monocyte/mesodermal lineage. Microglia are heterogeneously distributed in the adult brain, constituting between 5 and 12 % of cells depending on the region [111]. They represent an important component of the inflammatory response in the CNS in response to pathogens and injury [112]. In addition, microglia influence neurogenesis and synapse formation [113, 114]. When microglia are activated in response to a pathological change in the CNS, it can result in classically activated microglia (M1) and alternatively activated microglia (M2). M1 microglia are cytotoxic due to their release of proinflammatory cytokines, including IL-1β and TNFα, and reactive oxygen species (ROS). M2 microglia appear protective and release anti-inflammatory cytokines and neurotrophins, including IGF-1 [115]. Microglia have important functions in the protection and destruction of motor neurons [115–117]. There is increasing evidence that microglia are key components in ALS motor neuron degeneration [25, 117, 118]. mSOD1 motor neurons co-cultured with wild-type microglia did not develop disease, while mSOD1 microglia could induce wild-type motor neuron degeneration [16]. While reduction of mSOD1 in motor neurons delayed disease onset and early disease progression, diminished mSOD1 levels in microglia sharply slowed later disease progression. Thus, onset and progression represent distinct disease phases defined by mutant actions within different cell types to generate non-cell-autonomous killing of motor neurons [17, 18]. These findings validate therapies, such as cell replacement targeted to non-neuronal cells. In ALS pathology, there is an imbalance between the anti-inflammatory and neuroprotective roles of M2 microglia, in which they produce high levels of anti-inflammatory cytokines and neurotrophic factors, and their M1 cytotoxic responses [112, 115, 117]. fALS mouse models have an increased neuroprotective M2 microglial response in the initial phase of disease, but a mostly neurotoxic M1 response at end-stages [117], with production of NO depending on NOX2, pro-inflammatory cytokines (IL-6, IL12, IL-23, TNFα) and H_2_O_2_ ([117]; Fig. 1). Furthermore, in ALS, microglia appear to increase in cell size and granularity [30]. It has also been reported that microglia abnormally fuse to form multinucleated giant cells in spinal cord and brain and in later disease stages microglia showed cytoplasmic fragmentation, indicative of cellular dysfunction and degeneration [119]. Experimentation in fALS mice suggests that the replacement of disease-inducing microglia with healthy microglia through transplantation could represent an appealing approach to treat ALS in the future [26].

Microglia neuroprotection appears mainly induced by two signaling molecules, fractalkine and CD200, which are released from motor neurons. Fractalkine (CX3CL1) signaling promotes a dialogue between motor neurons and microglia, inducing microglia proliferation and chemotaxis; while, a lack of CX3CL1 induces neurotoxicity [120]. The cleaved form of this protein is released from motor neurons under stress conditions and binds to the fractalkine receptor (CX3CR1), which seems to be expressed by microglia. CX3CR1^−/−^ mice show more extensive neuronal degeneration than littermate controls, which is associated with microglia dysregulation, and studies with these mice confirmed the neuroprotective role of the CX3CL1/CX3CR1 interaction [120]. CX3CR1/GFP bone marrow cells could migrate into the CNS after transplantation and acquire positions close to blood vessels. This migration appeared more extensive in fALS mice than in wild-type littermates [121], suggesting that motor neuron degeneration (either directly or through increased inflammation) can attract cells from the outside (Table 2). However, most of the transplanted CXCR1 cells in the spinal cord appeared to associate with blood vessels, representing perivascular microglia (the cells located between glia and endothelial cells) rather than parenchymal microglia [121]. The CX3CR1 cells were partially immature at the time of transplantation; thus, the host environment could have influenced their differentiation into perivascular microglia rather than parenchymal microglia. It may be interesting to further differentiate the cells in vitro prior to transplantation to obtain committed parenchymal microglia, which are more involved in the ALS pathogenesis. CD200 (OX2) signaling also appears to play a critical role in ALS pathogenesis [121, 122]. This neuronal glycoprotein binds to the CD200 receptor (CD200R), which is expressed by all myeloid cells. Microglia in CD200^−/−^ mice undergo morphological changes from small cell bodies with numerous ramifications to larger cells with shorter processes. In addition, microglia switch from anti-inflammatory and neuroprotective to pro-inflammatory and neurotoxic, releasing ROS (NO_2_O_2_-, H_2_O_2_) and pro-inflammatory cytokines (IL-6; IL-12, IL-23, TNFα), further enhancing cell injury and initiating a self-propagating cycle of cell death ([122, 123]; Fig. 1). These modifications are not observed in CX3CR1^−/−^ mice, suggesting different protective roles for CD200/CD200R and CX3CL1/CX3CR1 in microglia control. It could be interesting to assess if overexpression of a molecule that inhibits microglia activation, such as CXCR1 or CD200R, can make donor microglia resistant to the degenerative environment in ALS.

**Table 2:** Recent advancements in bone marrow-derived cell (BMC) transplantation for ALS

Transplanted cells	Host	Outcome	References
Transplantation in rodent			
GFP/Thy1-YFP mice BMCs	SOD1^G93A^ mice	Delayed disease onset	[119]
		Increased life span	
		Decreased loss of motor neurons	
mSOD1^G93A^ mouse BMCs	PU.1^−/−^ mice	No clinical signs of ALS	[19]
Wild-type mouse BMCs	mSOD1^G93A^/PU.1^−/−^ mice	Benefits on disease course and survival	[17]
Wild-type mouse BMCs	Myelo-ablated SOD1^G93A^ mice	No benefit on disease progression	[112]
BMCs	SOD1^G93A^/CD4^−/−^ mice	Increased SOD1^G93A^ life-span	[20]
c-kit+ BMCs	SOD1^G93A^ mice	Reduced neuron loss and microgliosis	[111]
		Increased expression of GLT1	
		Benefit on disease course	
mSOD1 c-kit+ cells	SOD1^G93A^ mice	Increased neuron loss and microgliosis	
		No benefit on disease course	
Transplantation in humans			
HLA-matched HSCs	Six ALS patients (after total body irradiation)	Variable grade of engraftment in spinal cord	[113]
		No benefit on disease course	

Microglia can be artificially activated by LPS, which induces a switch from a protective to pro-inflammatory state with the production of IL-12, TNFα, NO, superoxide anions, and peroxynitrite (H_2_O_2_), causing motor neuron degeneration and exacerbation of disease progression in fALS mice [124–126]. These molecules promote interactions between extracellular glutamate and its receptor on motor neurons, resulting in higher levels of calcium entering the cells and inducing cell death cascades [122]. Due to the sensitivity of microglia to toxic signals, it must be considered that healthy, transplanted microglia may be negatively influenced by the host environment and thus contribute to an increase in pathological events, rather than the intended decrease.

A recent study showed that inflammatory monocytes were activated and their progressive recruitment to the spinal cord correlated with neuronal loss; while, resident microglia in the spinal cord decreased with disease progression. Prior to disease onset, splenic Ly6Chi monocytes showed a polarized macrophage phenotype with increased levels of chemokine receptor-2. As disease onset neared, microglia expressed increased chemokine (C–C motif) ligand 2 and other chemotaxis-associated molecules, which are involved in the recruitment of monocytes to the CNS by spinal cord–derived microglia. Anti-Ly6C mAb treatment reduced monocyte recruitment to the spinal cord, slowed down neuronal loss, and increased survival, suggesting that monocytes and microglia play an important role in ALS disease progression [127]. However, it is important to consider that many chimeric mouse ALS studies use whole-body irradiation as the regimen for bone marrow transplantation, which appears to cause disruptions in the BBB and thus could allow blood-derived monocytes to more freely enter the CNS and mature into microglia. Analysis of microglia progenitor recruitment from the circulation into the CNS using chimeric mice, obtained by parabiosis, indicated that recruitment from the periphery was very limited, even in mSOD mice [128]. While the parabiotic model could underestimate the recruitment of circulating microglia precursors to the CNS it is still a striking finding, which could indicate that circulating monocytes are being given a greater importance in ALS disease pathogenesis than they deserve. Several studies indicate that endogenous microglia proliferation in the lesioned CNS with intact BBB could account for increased Iba1+ cells [128–130]. Furthermore, microglia engraftment in the CNS, after bone-marrow transplantation, appeared to require conditioning of the brain with for example irradiation, if the BBB remained intact [130]. While there are several reports suggesting that the BBB shows disruption in ALS [131–133], the studies mentioned above indicate that transplantation of monocytes/microglia might require additional conditioning of patients for optimal recruitment and engraftment.

In vitro analysis, using COS-7 cells [134] and motor neuron-like NSC-34 cells [135] has shown that mSOD1 can be secreted, and in vivo analysis appears to support these observations [134], but extracellular mSOD1 alone does not appear to be directly toxic to motor neurons [126]. However, mSOD1 induces morphological and functional activation of microglia and when motor neurons are co-cultured with microglia, extracellular mSOD1 injures motor neurons. This activation of microglia by mSOD1 appears mediated through Toll-like receptor 2 (TLR-2), TLR-4, and CD14 [126]. Indeed, blocking CD14 results in reduced production of pro-inflammatory cytokines and free radicals and an increase in IGF-1 release from mSOD1^G93A^ microglia. Furthermore, microglia-mediated motor neuron toxicity is reduced in the presence of antibodies against TLR-2 and TLR-4, co-receptors of CD14 [126]. Expression of CD14 (as well as CD68) is increased in the spinal cords of ALS patients and in mSOD1 mouse models [118, 136], indicating that mSOD1 activation of microglia through CD14 could take place in vivo. These data seem to define a possible mechanism where extracellular mSOD1 protein acts in a LPS-like fashion and links to CD14, which in turn activates a pro-inflammatory cascade mediated by TLR2 and TLR4, reducing the action of neurotrophic factors. Thus, CD14 could be a possible molecular target for ex vivo genetic modification prior to cell transplantation to increase the therapeutic impact of microglia/hematopoietic cell transplantation.

The effects of microglia cell replacement by bone marrow-derived cells in ALS rodent models have been studied extensively with promising results. Transplantation of mSOD1^G93A^ microglia into PU.1^−/−^ mice, which lack macrophages, neutrophils, T- and B-lymphocytes, and microglia, did not induce motor neuron degeneration, confirming that mSOD1 in microglia alone is not sufficient to initiate disease ([17, 18, 35]; Table 2). When PU.1^−/−^ mice were bred with SOD1^G93A^ fALS mice and transplanted with wild-type bone marrow, motor neuron loss was slowed and disease duration prolonged compared with untransplanted mSOD1^G93A^ mice or mice receiving mSOD1^G93A^ cells ([126]; Table 2). The author also observed that transplanted cells differentiated mostly into microglia, while there were no identifiable astrocytes [126]. Consequently, the lack of mSOD1 in microglia may contribute to motor neuron protection and transplanting wild-type microglia could be beneficial to patients. Another important observation is that neuroprotection is not due only to microglia in the spinal cord; peripheral engraftment could also be important [126].

Once transplanted, bone marrow stem cells are able to generate mature CNS microglia that increase neuroprotective functions [26, 27, 122]. However, the extent of endogenous microglia replaced can depend on the transplantation protocol. Not surprisingly, there seems to be a correlation between the number of transplanted cells and therapeutic benefit [26, 27, 37, 83, 122]. On the other hand, differences in effects could also depend on technical aspects of the protocols, including the effect of irradiation in facilitating the migration of cells by BBB disruption and the cellular subtype composition of heterogeneous bone marrow transplants. For instance, a marked positive effect was observed when a specific c-kit+ bone marrow population was transplanted into SOD1^G93A^ mice ([137]; Table 2). Transplanting unmodified bone marrow–derived cells into 70-day-old SOD1^G93A^ mice resulted in spinal cord engraftment but had no effect on mouse survival ([26, 138]; Table 2). These results are comparable to data obtained in a clinical study based on HSCs intravenously administered into irradiated sALS patients. In that study, transplanted HSCs infiltrated areas characterized by damaged cells and neuroinflammation, acquiring an immunomodulatory cellular phenotype, but ALS patients did not show any clinical benefits ([139]; Table 2). Postmortem analysis showed large variations in engraftment, with a satisfactory degree of engraftment in some patients and no transplanted cells in others [139]. The majority of engrafted donor cells were macrophage-monocytes (CD68 positive), which localized to the lateral spinal motor columns. A low percent were leukocytes (CD45 positive) or CD8^+^ T cells, particularly around blood vessels [139]. Based on current data, insufficient engraftment is likely a key reason for the lack of benefit in transplanted patients. Pre-selection and enrichment of specific cellular populations with expected therapeutic benefits prior to transplantation and optimizing cell survival post-transplantation is of great clinical interest. A better understanding of the mechanisms involved when transplanted HSCs lead to a delay in disease in ALS mice could further clarify factors needed for graft benefit.

Based on the microglia transplantation approaches described (Table 2), it is appealing to hypothesize that bone marrow or hematopoietic cells could be modified ex vivo to express factors important for modulating an inflammatory response (such as IL-4, IGF-1, BDNF, GDNF) and/or blocking TLR/CD14 and then transplanted into ALS patients, taking advantage of the cells ability to migrate in the CNS (Fig. 3). In addition, it could also be interesting to modify the miRNA profile of these cells because they appear to play an essential role in the regulation of immune function [140]. The inflammatory miRNA signature in human ALS monocytes appears identical to that observed in SOD1^G93A^ mice, providing a correlation between mouse models and human disease [127]. This profile may be helpful as a biomarker of disease progression, but also to increase the therapeutic potential of these cells after transplantation. It will be very interesting to evaluate if microglia replacement, through modified cell transplantation, could positively influence the dysfunctional dialogue between host motor neurons and microglia.

## T-lymphocytes

T-lymphocytes play important roles in the non-cell autonomous mechanism that characterizes motor neuron damage in ALS. Therefore, in addition to astrocytes and microglial cell replacement, the transplantation of T lymphocytes may help normalize the delicate balance between neuroprotection and neurotoxicity. T lymphocytes are a type of white blood cell. They are produced in the bone marrow, complete their differentiation, mature in the thymus, and play a central role in cell-mediated immunity. They can be distinguished from other lymphocytes, including B cells and natural killer cells, by the presence of the T cell receptor (TCR) on their cell surface. There are several subsets of T-lymphocytes, each with a unique function. T-helper cells or CD4^+^ T-cells, express the CD4 protein on their surface and become activated when the TCR and CD4 bind to a peptide:major histocompatibility complex (MHC) II on antigen-presenting cells (APCs). The antigens presented are derived from extracellular proteins that are endocytosed, cleaved, bound to the MHC II in the cytosol, and finally presented on the cell surface. Once CD4^+^ T-lymphocytes are activated, they rapidly divide and start secreting a number of cytokines and induce maturation of B cells into antibody-producing plasma cells and memory B cells; in addition, they activate cytotoxic CD8^+^ T-lymphocytes and macrophages. CD8^+^ T-lymphocytes (or cytotoxic T-cells) destroy virally infected cells and cancer cells and are also implicated in transplant rejection. The CD8^+^ T-cells recognize intracellularly produced antigens presented by the MHC I, which is present on all nucleated cells in the body. When CD8^+^ T-lymphocytes become activated, they proliferate and differentiate into memory T cells and effector T cells, which secrete perforins and granzymes that are detrimental to the cells presenting antigens through MHC I.

In human autopsy material from ALS patients, there is significant accumulation of CD4^+^ and CD8^+^ T-lymphocytes in the spinal cord along with activated microglia, astrocytes, and deposits of IgG, presumably produced by plasma cells ([24, 33, 141, 142]; Fig. 1). These findings provide pertinent information about immune reactions at the end-stage of disease. To understand the role of immune cells, including T-lymphocytes, in earlier events, prior to onset, at onset, and during disease progression, studies in animal models of ALS are of vital importance. Interestingly, in SOD1^G93A^ ALS mice there was massive infiltration of CD4^+^ and CD8^+^ T-cells in the spinal cord just following the onset of symptoms [30, 143], indicating that T-lymphocytes are involved in earlier disease events. There was also striking microglial activation at the same time point ([18, 35, 143]; Fig. 1). Furthermore, deposits of IgG, IgM, and complement were detected in the sciatic nerves of SOD1^G93A^ mice along with macrophage accumulation [144].

Transplantation of bone marrow that contained red blood cells, platelets, and white blood cells including T and B cells in SOD1^G93A^ mice delayed the time of onset and increased life span, indicating that one or more of the cells present in bone marrow were beneficial in ALS ([144]; Table 2). To understand the contribution of T-lymphocytes to disease, SOD1^G93A^ mice were bred with mice lacking functional T-lymphocytes. Interestingly, breeding SOD1^G93A^ mice with recombinant-activating gene 2 (RAG2^−/−^) knock-out mice, which lack functional T-and B-lymphocytes, did not affect disease onset, but decreased the life-span and disease duration of the ALS mice, suggesting that T or B lymphocytes contribute to protection [27]. To better understand the involvement of B or CD4 T-lymphocytes in disease, SOD1^G93A^ mice were bred with CD4 knockout (CD4^−/−^) mice lacking surface expression of CD4, but with unaltered myeloid cells and CD8^+^ T- and B-lymphocytes. The resulting SOD1^G93A^/CD4^−/−^ mice did not have an altered disease onset, but had a shorter survival span and disease duration analogous to SOD1^G93A^/RAG2^−/−^ mice, indicating that CD4^+^ T cells were responsible for the prolonged disease duration and survival in SOD1^G93A^ mice. Interestingly, SOD1^G93A^/RAG2^−/−^and SOD1^G93A^/CD4^−/−^mice showed less activation of microglia at the end-stage compared to SOD1^G93A^/RAG2^+/−^or SOD1^G93A^/CD4^+/−^ mice, even though their survival time was shorter. However, mRNA levels of neurotrophic factors, including IGF-I, GDNF, BDNF, and anti-inflammatory factors were decreased in the spinal cords of SOD1^G93A^/RAG2^−/−^and SOD1^G93A^/CD4^−/−^mice, as were the levels of CX3CR1 and several glutamate receptors. Bone marrow transplants restored the levels of these substances, indicating that the presence of CD4 T-lymphocytes alter microglial and astroglial activation in ALS and may support motor neuron protection by modulating the glial balance between trophism and cytotoxicity [27]. This is consistent with previous findings demonstrating that T-lymphocytes can modulate microglial activation and provide neuroprotection in acute models of neuronal injury [145–148] and shows that controlled immune activation could be beneficial for regenerative processes. T-cells also brought about a neuroprotective response in spinal cord microglia [27, 30, 143]. Since a large population of motor neurons and axons remain intact at symptom onset, the role of the immune system might be to protect these remaining neurons from degeneration. Interestingly, in Parkinson disease CD4^+^ T lymphocyte infiltration appears deleterious, as shown in a model where dopamine neuron degeneration in response to the neurotoxin 1-methyl-4-phenyl-1,2,3,6-tetrahydropyridine required T lymphocyte infiltration, and seemed to involve a Fas/Fas-ligand-dependent mechanism [149].

Recently, studies have shown that regulatory CD4(+)CD25(high) T lymphocytes (Tregs) and cytotoxic CD4(+)CD25(−) T lymphocytes (Teffs) have distinct roles in the pathology of ALS [125]. mSOD1 Tregs co-cultured with mSOD1 adult microglia repressed cytotoxic microglial factors such as NOX2 and iNOS through an IL-4-mediated mechanism. On the other hand, Teffs were only modestly effective in repressing microglia toxicity [28, 150]. The roles of these cellular types appear to differ during the different stages of pathology. During the earlier, stable disease phase, the numbers of Tregs increased, specifically those that were IL-4^+^, IL-10^+^, or TGF-β^+^. Tregs isolated from this disease stage inhibited Teff proliferation through the combined action of IL-4, IL-10, and TGF-β. On the other hand, during the rapidly progressing, later disease phase, the number of mSOD1 Tregs diminished, while the proliferation of mSOD1 Teffs increased and was not suppressed by IL-4, IL-10, and TGF-β. Consequently, mSOD1 Tregs contributed to slowing down the progression phase in ALS mice and may offer a novel therapeutic option for ALS patients in the stable disease phase [28, 29]. Without ex vivo activation, the passive transfer of mutated SOD1 Tregs from early disease ALS mice into ALS mice was more immunotherapeutic than the passive transfer of wild-type CD4^+^ T cells. The stable disease phase was extended by 88 % and prolonged survival was observed [27]. These effects could be mediated by the augmented secretion of IL-4 from mutant SOD1 Tregs, that directly suppressed the toxic properties of microglia. The data obtained in ALS mice models are consistent with data from the ALS patient population with the numbers of Tregs inversely correlating with disease progression rates [28, 29]. The identical findings in mouse and human ALS suggest that increasing the levels of regulatory T lymphocytes in ALS patients at early stages may have potential therapeutic value, and could aid in stabilizing patients for longer periods of time.

What is causing the activation of T-lymphocytes during the progression of disease in ALS? Some possibilities are events within motor neurons, microglia, and astrocytes or antigen-presentation on APCs. Interestingly, components (C1qa, C1qb, C1qc) of the classical complement pathway were induced in or closely surrounding motor neurons in response to overexpression of disease-causing mSOD1. The C1q induction occurred prior to the appearance of obvious clinical symptoms or major neuroinflammation and could contribute to neurodegeneration [151]. C1q is a secreted extracellular polypeptide, which can bind antibody aggregates. It is the main initiating factor for the classical complement system, which is used to clear/lyse pathogens in injured or degenerating cells [151]. Motor neurons overexpressing mSOD1 secreted a proportion of the mSOD1 protein [134], which might be recognized by the C1q-induced complement system and mark the motor neurons for attack by immune cells. Furthermore, this secreted mSOD1 could be taken up by APCs presented by the MHC II on their surface and subsequently cause the activation and proliferation of CD4^+^ T-cells. This can also cause the activation of B cells and differentiation into plasma cells, producing large quantities of antibodies against mSOD1 and other misfolded proteins that would deposit in tissues causing further damage. Interestingly, the complement system also impacts T-cell immunity during the induction, effector, and contraction phases of an immune response. These modulatory effects of complement on T-cell responses were mediated by inducing specific signaling events in the T-cell itself and indirectly through alterations of APCs [152]. Moreover, the complement system is involved in an adaptive response in B and T cell immunity and required for cell-mediated immunity elicited through CD4^+^ T-lymphocyte producing interferon gamma as an effector molecule (a Th1 response).

Importantly, expanding the T-lymphocyte population by transplanting Tregs may offer further protection in ALS and could provide a novel therapeutic target. Additional T-lymphocytes could modify the dysfunctional interaction between host astrocytes, microglia, and motor neurons. Engineering T-lymphocytes to express factors important for modulating an inflammatory response (such as IL-4, IL-10, and TGF-β), to express neurotrophic factors (BDNF, GDNF, IGF-1), or to silence pro-inflammatory factors such as TNF-α and especially Nox2 could even further stimulate neuroprotection (Fig. 3).

Recently, it was demonstrated that neuronal stem cell (NSC) transplantation could ameliorate pathological mechanisms in the mutant SOD1^G93A^ mouse model of ALS. These cells appeared to produce trophic factors, preserve neuromuscular function, and reduce astrogliosis and inflammation [153]. A phase I clinical trial was initiated at Emory University in 2010 to assess the safety and tolerability of intraspinal injections of fetal human spinal cord-derived NSCs in 18 ALS patients (http://www.alsconsortium.org/trial.php?id=12; [154]). According to an initial report from this trial, all patients tolerated the treatment without any long-term complications related to the surgical procedure or stem cell transplantation. There was no evidence of acceleration of disease following stem cell injections. It is possible that some patients may have slowed their lower limb progression, but a larger trial needs to be conducted to properly evaluate this [154]. Subsequently, a phase II clinical trial using fetal-derived spinal cord NSC in 15 ALS patients to assess safety and benefits, to be conducted at Emory University, was just approved by the USA Food and Drug Administration (http://www.neuralstem.com/cell-therapy-for-als). Patients will receive multiple injections in the cervical spinal cord with the goal of preserving respiratory motor neuron pools. Concomitantly, a phase I clinical trial using NSCs derived from fetal cortices, genetically engineered to overexpressed the trophic factor glial cell line-derived neurotrophic factor (GDNF) [155], will be conducted through California’s stem cell agency (http://www.cirm.ca.gov/our-funding/awards/progenitor-cells-secreting-gdnf-treatment-als). The transplantation of SOD1^G93A^ fALS rats using a similar approach protected the integrity of motor neuron cell bodies, but failed to preserve neuromuscular junctions or increase the life-span of the animals [155].

## Conclusions

At this time, no cure or efficacious treatments are available for ALS and the major pathogenic mechanisms underlying the selective motor neuron degeneration are still largely unknown. While initiation of motor neuron degeneration appears cell-intrinsic, other cell types including astrocytes, microglia, and T-lymphocytes are key components in the disease progression of ALS, and cell transplantation could more effectively be targeted at replacing these cell types rather than motor neurons.

These cellular populations have shown therapeutic potential, particularly in moderating inflammatory responses after transplantation into fALS mouse models, and may present new avenues for the treatment of this disorder (Fig. 3). For treatments, it would be beneficial to have a cellular source that can replicate in vitro to generate large numbers of cells and can then be coaxed into generating specific cell types, without forming tumors, in vivo. After transplantation, these cells should migrate to sites of motor neuron degeneration and replace the host’s toxic support cells in close proximity to motor neurons and promote motor neuron survival and function. To optimize the therapeutic benefit of such cellular transplantation aimed at preventing further neurodegeneration, but without restoration of motor neuron numbers, it needs to be initiated as early as possible in the disease course. Furthermore, to increase the therapeutic impact of cellular therapy, genetic modifications of donor cells may be necessary. If transplanted cells could migrate longer distances into appropriate sites, their therapeutic potential would greatly increase in this multifocal disease. Furthermore, donor cells need an enhanced resistance to the toxic disease environment they will encounter. Finally, transplanted cells should have an improved ability to promote motor neuron protection, by either secreting protective factors or sequestering toxic substances from the surroundings.

Recent advances in stem cell approaches have led to the generation of unlimited numbers of cells, including neurons, astrocytes, and microglia, both heterologous and autologous. However, patient-specific cells should be treated with some caution, since these likely maintain disease predisposition after reprogramming and, thus, could be toxic. In terms of heterologous transplantation, it might be necessary to use immunosuppression to avoid transplant rejection. Such treatment would have effects on neuroinflammation and adaptive immunity triggered by the disease itself, which could affect the disease outcome. However, clinical trials from Parkinson disease using fetal dopamine neurons have shown that transplanted cells can survive in the brain without immunosuppression [156]. Thus, it is likely that also in ALS, immunosuppression can be omitted, or at least kept to a minimum, should this be beneficial for the patient. To facilitate a safe and practical application in humans, intrathecal or systemic injections should be assessed and implemented in pre-clinical models. In the future, an ideal cell therapy could include the transplantation of combinations of cellular subtypes, motor neurons, immune cells, or astrocytes, with the aim of addressing both the autonomous and non-autonomous cellular components for an effective ALS therapy.

## Abbreviations

ALS: Amyotrophic lateral sclerosis
ANG: Angiogenin
APS: Antigen-presenting cells
BLBP: Brain lipid binding protein
CHG: Chromogranin
CNS: Central nervous system
CCL2: Chemokine (C–C motif) ligand 2
CCR2: Chemokine receptor-2
DRG: Dorsal root ganglia
EAAT: Excitatory amino acid transporter
fALS: Familial amyotrophic lateral sclerosis
FGF-1: Fibroblast growth factor 1
FUS/TLS: Fused in sarcoma/translocated in liposarcoma
GDNF: Glial cell line-derived neurotrophic factor
GFAP: Glial fibrillary acidic protein
GRPs: Glial-restricted precursors
HSC: Hematopoietic stem cells
LPS: Lipopolysaccharide
MHC: Major histocompatibility complex
mSOD1: Mutant superoxide dismutase 1
NGF: Nerve growth factor
NSC: Neuronal stem cell
OPTN: Optineurin
PFN: Profilin
PSA-NCAM: Polysialylated neural cell adhesion molecule
RC2: Radial glial cell marker-2
sALS: Sporadic amyotrophic lateral sclerosis
SDF-1α/CXCR4: Stromal cell-derived factor 1α/chemokine receptor 4
TDP-43: TAR DNA-binding protein 43
Teffs: CD4(+)CD25(−) T lymphocytes
TLR: Toll-like receptor
Tregs: CD4(+)CD25(high) T lymphocytes
VCP: Valosin-containing protein
